# Efficacy and safety of Shugan Jieyu Capsule in the treatment of depressive state after acute coronary syndrome: study protocol for a multicenter randomized controlled trial

**DOI:** 10.3389/fpsyt.2025.1683736

**Published:** 2025-10-16

**Authors:** Yankai Yang, Zhuorui Cui, Furong Yang, Yanqiao Yu, Yajie Cai, Xiaodi Fan, Qiaoning Yang, Ruina Bai

**Affiliations:** ^1^ Xiyuan Hospital, China Academy of Chinese Medical Sciences, Beijing, China; ^2^ National Clinical Research Center for Chinese Medicine Cardiology, Beijing, China; ^3^ Beijing University of Chinese Medicine, Beijing, China; ^4^ Department of Traditional Chinese Medicine, Beijing Hospital, Beijing, China

**Keywords:** Shugan Jieyu Capsule, acute coronary syndrome, depressive state, randomized controlled trial, protocol

## Abstract

**Objective:**

This study aims to evaluate the efficacy and safety of Shugan Jieyu Capsules (SGJY) in patients with depressive state after Acute Coronary Syndrome (ACS).

**Methods:**

This is a multicenter, randomized, double-blind, placebo-controlled clinical trial. A total of 148 patients with depressive state after ACS recruited from five research centers, will be randomly assigned to either the SGJY group or the placebo group at a 1:1 ratio. In addition to standard therapies for ACS, the SGJY group will receive SGJY while the placebo group will receive a matching placebo. All participants will undergo 12 weeks of treatment, followed by 36 weeks of follow-up.

**Results:**

The primary outcome is the Hamilton Depression Rating Scale (HAMD-17). Secondary outcomes include major adverse cardiovascular and cerebrovascular events (MACCE), Seattle Angina Questionnaire (SAQ) score, depression and anxiety scales, the Short Form-36 (SF-36) health survey, Montreal Cognitive Assessment (MoCA) score, inflammatory cytokine levels, hypothalamic-pituitary-adrenal (HPA) axis activity, and brain-derived neurotrophic factor (BDNF) levels. Safety will be evaluated based on safety indicators and recorded adverse events. Additionally, metabolomic analysis will be conducted on patient serum samples collected before and after treatment to elucidate the potential metabolic pathways of SGJY ameliorates subthreshold depression after ACS.

**Conclusion:**

This trial will evaluate the efficacy and safety of SGJY in managing depressive state after ACS. Additionally, the potential of SGJY to improve long-term prognosis in patients with depressive state after ACS will be assessed.

## 1 Introduction

Cardiovascular disease remains the leading cause of death worldwide, with acute coronary syndrome (ACS) being one of the most prevalent and life-threatening manifestations (1). Despite administering secondary prevention according to guidelines, the risk of recurrent coronary events remains as high as 32.5% within 2 years after discharge, with a 5-yearcardiovascular mortality rate of 11.2% (2). Notably, ACS-related hospitalizations account for the largest proportion (38.6%) of total cardiovascular-related healthcare expenditures (3). Furthermore, studies have shown that over 20% of patients experience depression after acute myocardial infarction (4), which increases the risk of major adverse cardiovascular events by 51% (5) in individuals with coronary artery disease, imposing a substantial economic burden on both families and society (6).

The American Heart Association (AHA) and European Society of Cardiology (ESC) guidelines recommend routine depression screening and appropriate therapeutic management for all individuals with cardiovascular disease (7, 8). While selective serotonin reuptake inhibitors (SSRIs) are considered first-line pharmacological agents for treating depression after ACS, their ability to reduce cardiovascular adverse events in patients with comorbid ACS and depression remains uncertain (9, 10). In addition, long-term use of SSRIs may cause varying degrees of side effects and drug resistance (11, 12), leading to reduced quality of life and poor treatment adherence.

Traditional Chinese medicine (TCM), known for its multi-component, multi-target, and multi-pathway synergistic effects, is commonly used as an adjunctive therapy. Shugan Jieyu Capsules (SGJY), the first Chinese patent medicine approved in China for the treatment of mild to moderate depression, is a nationally second-class protected TCM formulation composed of two herbs: *Hypericum perforatum* and *Acanthopanax senticosus*. Existing clinical evidence suggests that SGJY may alleviate symptoms in patients with coronary heart disease comorbid depression, with relatively mild adverse effects. However, the overall quality of the evidence remains low, as it primarily focuses on changes in depression rating scales while devoting limited attention to cardiac function and long-term outcomes (13). Furthermore, most previous clinical studies have focused on changes in depression rating scales. To address this gap, we designed a multicenter, randomized, double-blind, placebo-controlled clinical trial to assess the effects of SGJY on patient with depressive state after ACS. Specific efficacy indicators including quality of life, long-term cardiac prognosis, cardiopulmonary function, serum inflammatory markers, hypothalamic-pituitary-adrenal (HPA) axis-related hormones, and brain-derived neurotrophic factor (BDNF). The study will also explore the underlying mechanisms of SGJY in treating depressive state after ACS through metabolomic analysis.

## 2 Materials and methods

### 2.1 Design and settings

This is a multicenter, randomized, double-blind, placebo-controlled clinical trial. The trial was registered on the National Medical Research Registration and Filing Information System (www.medicalresearch.org.cn) on June 19, 2024 (Registration No. MR-11-24-037476), and on the International Traditional Medicine Clinical Trial Registry (itmctr.ccebtcm.org.cn) on June 1, 2024 (Registration No. ITMCTR2024000099). All documents, including the study protocol, informed consent forms, and case report forms (CRFs), comply with the Declaration of Helsinki and have been reviewed and approved by the Xiyuan Hospital Ethics Committee of the Chinese Academy of Medical Sciences (approval number: 2024XLA062-3). The clinical trial protocol follows the SPIRIT 2025 statement (14). The trial will be conducted in accordance with the Good Clinical Practice (GCP) Guidelines (2020) and reported according to the CONSORT 2025 statement (15).

**Figure 1:**
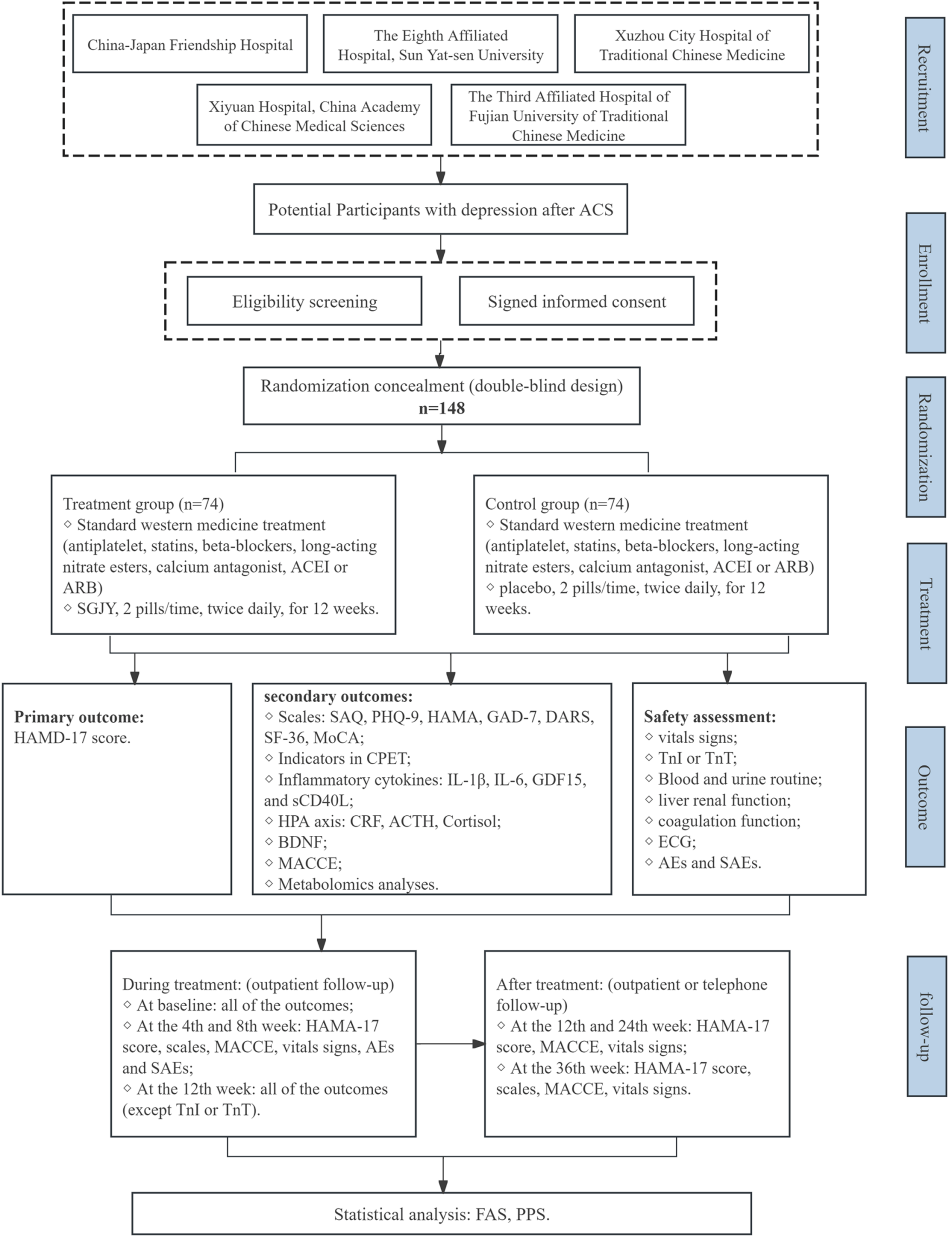
Flow chart of the study design. ACS, Acute Coronary Syndrome; ACEI, angiotensin-converting enzyme inhibitor; ARB, angiotensin receptor antagonist; SGJY, Shuganjieyu Capsule; HAMA-17, Hamilton depression rating scale; MACCE, Major adverse cardiovascular and cerebrovascular events; SAQ, Seattle angina questionnaire; PHQ-9, Patient health questionnaire-9; HAMA, Hamilton Anxiety Scale; GAD-7, Generalized anxiety disorder-7; DARS, Dimensional Anhedonia Rating Scale; SF-36, Short form 36 health survey; MoCA, Montreal cognitive assessment; CPET, cardiopulmonary exercise testing; IL-1β, Interleukin 1 beta; IL-6, Interleukin 6; GDF15, Growth differentiation factor 15; sCD40L, Soluble CD40 ligand; CRF, Corticotropin-releasing factor; ACTH, Adrenocorticotropic hormone; BDNF, Brain-derived neurotrophic factor; TnI, Troponin I; TnT, Troponin T; ECG, Electrocardiogram; AEs, Adverse events; SAEs, Severe adverse events; FAS, Full analysis set; PPS, Per protocol set; SAS, Safety analysis set.

Patients will be recruited from five centers in China: Xiyuan Hospital, China-Japan Friendship Hospital, the Eighth Affiliated Hospital of Sun Yat-sen University, the Third People’s Hospital of Fujian Province and Xuzhou City Hospital of TCM. A total of 148 participants will be enrolled after providing written informed consent. Participants will be randomly assigned in a 1:1 ratio to receive either SGJY or placebo, with a 12-week treatment period followed by a 36-week follow-up. A schematic diagram of the study procedures is presented in **Figure 1**.

### 2.2 Study population

#### 2.2.1 Diagnostic criteria

The diagnostic criteria for ACS derived from the *2023 ESC Guidelines for the Management of Acute Coronary Syndromes* (16), while the diagnostic criteria for depressive state is drawn from the *Expert Consensus on the Diagnosis and Treatment of Anxiety, Depression, and Somatization Symptoms in General Hospitals* (17).

#### 2.2.2 Inclusion criteria

Patients who eligible for the trial must fulfill all of the following criteria at the time of randomization:

1. Participants are aged between 18 and 75 years.
2. A confirmed diagnosis of ACS within 2 to 12 weeks prior to enrollment, with hemodynamic stability and absence of acute cardiovascular complications.
3. Participants exhibited persistent symptoms of depressed mood or anhedonia for at least 2 weeks, with 17 < HAMD-17 ≤ 24
4. All eligible participants must provide written informed consent.

Note:

1. Depressed Mood: The affective manifestation persists nearly daily for the majority of waking hours, manifesting through either subjective affective experience (e.g., persistent feelings of sadness, emotional emptiness, or hopelessness) or objective behavior observed by others (e.g., frequent episodes of tearfulness).
2. Anhedonia: Present almost daily occurrence for most of the day. This involves a significant decrease in interest or pleasure in all or almost all activities (which can be either self-reported or observed).

#### 2.2.3 Exclusion criteria

Potential participants meeting any of the following criteria will be excluded from this trial enrollment:

1. Patients with impaired liver function (serum alanine aminotransferase or aspartate aminotransferase greater than 1.5 times the upper limit of normal value), impaired renal function (serum creatinine or blood urea nitrogen greater than the upper limit of normal value);
2. Patients with severe hematologic disorders, malignant tumors, malignant arrhythmias, pulmonary edema, severe aortic valve stenosis, severe anemia;
3. Patients with a history of depression or psychiatric disorders preceding the onset of ACS, or with marked suicidal ideation;
4. Patients with heart failure classified as New York Heart Association (NYHA) class III-IV, left ventricular ejection fraction (LVEF) <35%, or those requiring mechanical circulatory support;
5. Patients who have taken antidepressants or medications containing *Acanthopanax senticosus* or *Hypericum perforatum* chronically or within the past 4 weeks;
6. Pregnant or lactating women, or those planning to conceive;
7. Patients with autoimmune diseases (e.g., rheumatoid arthritis, ankylosing spondylitis, and inflammatory bowel disease) or taking immunosuppressive drugs within the last three months;
8. Patients with acute infections in the past 4 weeks, or those showing signs or symptoms of infection (e.g., fever, cough, dyspnea, leukocytosis);
9. Patients who have participated in or are currently enrolled in other clinical trials within the past 3 months;
10. Patients with known hypersensitivity to any component of the study drug.

#### 2.2.4 Termination and dropout of trial patients

1. Patients who fail to meet the inclusion criteria or fulfill any exclusion criteria.
2. Patients with poor protocol compliance or who withdrew prematurely from the trial.
3. Patients with incomplete medical records that hinder the evaluation of efficacy and safety.
4. Patients whose condition deteriorated rapidly during the study, including those with acute cardiovascular events or malignant neoplasms.
5. Patients whose depression worsens during the trial (HAMD-17 score >24 for over two weeks) or who display suicidal ideation.
6. Patients with serious adverse events during the trial.
7. Voluntary withdrawal by the patient.

#### 2.2.5 Handling of dropout cases

1. After a trial participant drops out, researchers should maintain contact with them through means including phone calls, scheduled check-ins, follow-up visits, or written correspondence, inquire about the reasons for dropout, document the last dose administration time, and complete any feasible assessments.
2. All selected trial participants, regardless of whether they drop out, should have their research-related medical records properly documented.

### 2.3 Randomization, allocation concealment, and blinding

#### 2.3.1 Random allocation

All eligible patients who provide informed consent will be randomly assigned in a 1:1 ratio to either the control group or the SGJY group. Stratified block randomization will be used, with clinical research center as the stratification factor. A statistician independent of trial analysis will generate the randomization table using SAS software, based on the stratification factor and allocation ratio. The number of blocks, block length, random seed parameters, and other confidential data will be sealed along with the randomization table in an opaque envelope, which will serve as the First unblinding record.

#### 2.3.2 Blinding

This study adopts a double-blind design. The first level blinding corresponds to the group codes linked to medication numbers (Group A, Group B), while the second level pertains to the treatments assigned to each code (SGJY group or placebo control group). Both levels of blinding will be documented by statisticians independent of trial analysis. Two sealed copies of the blinding records will be maintained separately by the principal research center. These blinding records must remain unopened throughout the study period. The first unblinding for the statistical analyst will conduct after data lock, and the second after completion of the statistical analysis. Emergency envelopes may only be opened in the event of a medical emergency (serious adverse event, rapidly deteriorating condition). Each envelope will contain designated fields for the signature, date, and reason for unblinding by the person who opens it. Cases withdrawn due to therapeutic reasons should not be unblinded. The unblinded case materials must be kept intact.

#### 2.3.3 Sample size calculation

The sample size was calculated based on the response rate of HAMD-17 score reduction, the primary efficacy endpoint. Based on existing literature, the response rate of HAMD-17 score reduction is 45.7% (18) in the control group and 69% in the Shu Gan Jie Yu group (19). Assuming a two-sided significance level (α) of 0.05 and power (1−β) of 80%. The ratio of the two sample sizes is 1. Thus, a sample size of 67 participants per group is required. Allowing for a maximum dropout rate of 10%, the sample size was increased to 74 per group, for a total enrollment of 148 participants.

### 2.4 Interventions

The investigational drug (SGJY) and placebo are capsule formulations provided by Sichuan Jishengtang Pharmaceutical Co., Ltd. (Pengzhou, China). The composition of SGJY includes *Hyperic perforated herba, Acanthopanax senticosi radix et rhizoma seu caulis* (**Table 1**). SGJY and placebo capsules are identical in packaging, color, shape, and flavor, ensuring that neither participants nor investigators can distinguish the group allocation prior to unblinding.

**Table 1:** The composition of Shuganjieyu Capsule (intervention drug).

Chinese name	Scientific name	Latin name	Species name
Guan ye jin si tao	Hypericum perforatum L.	*Hyperic perforated herba*	Garcinia
Ci wu jia	Acanthopanax senticosus (Rupr.et Maxim.) Harms	*Acanthopanax senticosi radix et rhizoma seu caulis*	Ginseng

#### 2.4.1 Standard medical management

In accordance with current ACS treatment guidelines (20), participants will receive standard therapy, including antiplatelet agents, statins, and anti-ischemic medications (e.g., β-blockers, long-acting nitrates, calcium channel blockers). The type and dosage of medications administered to each patient will be documented in the case report form (CRF).

#### 2.4.2 Experimental group

Participants in the experimental group will receive SGJY in combination with standard medical therapy. The dosage regimen is 0.36 g per pill, two pills orally, twice daily. The treatment period will last for 12 weeks. Participants will be instructed to return any unused medication and packaging at each visit to assess adherence.

#### 2.4.3 Control group

Participants in the control group will receive placebo capsules. The dosage regimen of the placebo will match that of the SGJY group.

#### 2.4.4 Drugs combined and contraindicated in trial

During the trial, the use of other traditional Chinese medicine (TCM) decoctions, oral or intravenous TCM patent medicines, or TCM-based therapies (e.g., acupuncture or cupping), as well as paroxetine, sertraline, and other drugs that may interfere with depressive state after ACS, will be prohibited. Adjunctive treatments for depression, such as psychological counseling and supportive psychotherapy, are prohibited as well. Concomitant treatment for comorbidities is permitted during the trial. However, all concomitant treatments must be documented in CRF, including the medication (or therapy) name, dosage, frequency, and treatment duration.

#### 2.4.5 Follow up

After enrollment, all participants will undergo seven follow-up assessments. Outpatient visits will be scheduled at weeks 4, 8, and 12 post-treatment, with a ±3-day window from the start of medication administration. Additionally, outpatient or telephone follow-ups will occur at weeks 12, 24, and 36 after completion of the medication regimen.

### 2.5 Outcome

The details of items to be measured and time points for data collection are shown in **Table 2**.

**Table 2:** Trial schedule.

Study phase		Screening period	Intervention period (12 weeks)			Follow-up period (36 weeks)		
Visit		V1	V2	V3	V4	V5	V6	V7
		-3–0 days	Week 4 ± 3 days	Week 8 ± 3 days	Week 12 ± 3 days	Week 24 ± 3 days	Week 36 ± 3 days	Week 48 ± 3 days
Informed consent		**×**						
Random allocation		**×**						
Inclusion/Exclusion Criteria examination		**×**						
Baseline data collection	Demographic information	**×**						
	Medical history	**×**						
	Past medical history, allergies	**×**						
Safety evaluation	Vital signs	**×**	**×**	**×**	**×**	**×**	**×**	**×**
	Blood and urine routines	**×**			**×**			
	Liver and renal function	**×**			**×**			
	coagulation function	**×**			**×**			
	ECG	**×**			**×**			
	TnI or TnT	**×**						
Outcome evaluation	HAMD-17	**×**	**×**	**×**	**×**	**×**	**×**	**×**
	SAQ	**×**	**×**	**×**	**×**			**×**
	PHQ-9	**×**	**×**	**×**	**×**			**×**
	HAMA	**×**	**×**	**×**	**×**			**×**
	GAD-7	**×**	**×**	**×**	**×**			**×**
	DARS	**×**	**×**	**×**	**×**			**×**
	SF-36	**×**	**×**	**×**	**×**			**×**
	MoCA	**×**	**×**	**×**	**×**			**×**
	CEPT	**×**			**×**			
	IL-1β, IL-6, GDF15, and sCD40L	**×**			**×**			
	HPA	**×**			**×**			
	BDNF	**×**			**×**			
	MACCE		**×**	**×**	**×**	**×**	**×**	**×**
	AE		**×**	**×**	**×**			
Investigational Medication Distribution		**×**	**×**	**×**				
Investigational Medication Collection			**×**	**×**	**×**			
Log Cards Issuance		**×**	**×**	**×**				
Log Cards Collection			**×**	**×**	**×**			
Record drug combination		**×**	**×**	**×**	**×**	**×**	**×**	**×**
CRF principal investigator examination								**×**
CRF monitor examination								**×**

HAMA-17, Hamilton depression rating scale; MACCE, Major adverse cardiovascular and cerebrovascular events; SAQ, Seattle angina questionnaire; PHQ-9, Patient health questionnaire-9; HAMA, Hamilton Anxiety Scale; GAD-7, Generalized anxiety disorder-7; DARS, Dimensional Anhedonia Rating Scale; SF-36, Short form 36 health survey; MoCA, Montreal cognitive assessment; CPET, cardiopulmonary exercise testing; IL-1β, Interleukin 1 beta; IL-6, Interleukin 6; GDF15, Growth differentiation factor 15; sCD40L, Soluble CD40 ligand; CRF, Corticotropin-releasing factor; ACTH, Adrenocorticotropic hormone; BDNF, Brain-derived neurotrophic factor; TnI, Troponin I; TnT, Troponin T; ECG, Electrocardiogram; AE, Adverse event.

#### 2.5.1 Patient characteristics

Demographic factors include age, gender, height, weight, ethnicity, educational level, socioeconomic status, and family history of hereditary diseases. Psychiatric history includes previous mental illness episodes and current or previous use of psychiatric medications. Lifestyle factors include smoking status, alcohol consumption, and physical activity levels. Cardiac history includes first occurrence status, ACS subtype, previous percutaneous coronary intervention (PCI) or coronary artery bypass grafting (CABG), myocardial injury biomarker abnormalities, and coronary artery condition. Comorbidities include hypertension, diabetes mellitus, hyperlipidemia, renal disease, hepatic disease, and other relevant conditions. This information will be collected at baseline and evaluated during the data management.

#### 2.5.2 The primary and secondary outcomes

##### 2.5.2.1 Primary outcome

The Hamilton Depression Rating Scale (HAMD-17) will be evaluated at each visit. The primary outcome is the proportion change in patients with a reduction rate more than 50% or a HAMD-17 score of less than 7 after 12 weeks of treatment. The reduction rate will be calculated using the following formula: [(baseline score - score at week 12)/baseline score] × 100%. The efficacy index is classified into three categories: cured (HAMD-17 score < 7), effective (reduction rate > 50%), and ineffective (reduction rate < 50%). The overall response rate is calculated as: [(cured cases + effective cases)/total cases] × 100%.

##### 2.5.2.2 Secondary outcomes

The secondary outcomes are as follows:

1. Major Adverse Cardiovascular and Cerebrovascular Events (MACCE), defined as a composite of cardiovascular death, nonfatal myocardial infarction, revascularization, and stroke. These events will be recorded throughout the study period. The cumulative incidence of MACCE will be analyzed over the 36-week follow-up period.
2. The Seattle Angina Questionnaire (SAQ) evaluates five health domains in patients with stable coronary artery disease: physical limitation, anginal stability, anginal frequency, treatment satisfaction, and disease perception.
3. The Patient Health Questionnaire-9 (PHQ-9), Hamilton Anxiety Scale (HAMA), Generalized Anxiety Disorder-7 (GAD-7) and Dimensional Anhedonia Rating Scale (DARS) will be administered at baseline, and at weeks 4, 8, and 12 post-treatment, as well as at the 36-week follow-up, to assess anxiety and depressive symptoms.
4. The Short Form-36 Health Survey (SF-36) will be administered at baseline, and at weeks 4, 8, and 12 post-treatment, as well as at the 36-week follow-up, to assess patients’ quality of life.
5. The Montreal Cognitive Assessment (MoCA) will be administered at baseline, and at weeks 4, 8, and 12 post-treatment, as well as at the 36-week follow-up, to evaluate cognitive function.
6. Improvement of Indicators in Cardiopulmonary Exercise Testing (CPET): (a) Metabolic Equivalents (METs), exercise time, Peak oxygen uptake (PeakVO2), oxygen pulse (VO2/HR), oxygen uptake relative to work rate (ΔVO2/ΔWR), and ventilatory anaerobic threshold (VAT). These parameters reflect exercise tolerance and cardiovascular function. (b) Breathing reserve (BR), the dead space volume/tidal volume ratio (VD/VT) and breathing frequency (Bf). These parameters reflect ventilatory function. (c) Partial Pressure of End-Tidal Carbon Dioxide (PETCO2), and the ventilatory equivalent of carbon dioxide (VE/VCO2 slope): These reflect the efficiency of pulmonary gas exchange. CPET includes over 50 indicators, each of which holds prognostic significance across cardiovascular conditions.
7. Inflammatory cytokines (IL-1β, IL-6, GDF15, and sCD40L) and hormones related to the HPA axis—corticotropin-releasing factor (CRF), adrenocorticotropic hormone (ACTH), and cortisol—will be measured. BDNF will also be assessed. Blood samples will be collected before treatment and on the day of the end-of-treatment.

#### 2.5.3 Safety outcomes

1. General physical examinations will be recorded at each visit point, including temperature, respiration, heart rate, and blood pressure.
2. Routine laboratory tests for blood and urine, liver and kidney function, coagulation function and electrocardiogram (ECG) will be conducted at baseline and at the end of treatment.

#### 2.5.4 Metabolomics analysis

Blood samples will be collected for metabolomic analysis. Liquid chromatography-mass spectrometry (LC-MS) and gas chromatography-mass spectrometry (GC-MS) will be used to identify chemical and biological fingerprints, screen bioactive components, and explore the potential metabolic pathways and targets of SGJY in the treatment of depressive state after ACS. Participants will be required to fast for at least 8 hours prior to blood collection. A 5 mL blood sample will be collected into heparin sodium anticoagulant tubes, centrifuged, and stored in Eppendorf tubes at −80°C.

#### 2.5.5 Adverse events

This study will monitor all adverse events (AEs), serious adverse events (SAEs), and adverse drug reactions (ADRs). All AEs must be promptly documented in both the source documents and the CRFs upon discovery. If an AE is classified as an SAE or a serious ADR, it must be reported in accordance with the applicable reporting procedures.

Due to the nature of the underlying diseases in this study, participants may experience clinical deterioration during the observation period, potentially requiring hospitalization or resulting in life-threatening events. However, MACCE is a predefined efficacy outcome in this study. Therefore, the occurrence of these events will not be documented as AEs. Likewise, hospitalizations or deaths resulting from these events will not be reported as SAEs.

All AEs must be followed until the participant’s condition returns to baseline (pre-treatment) levels or stabilizes. Investigators are responsible for continuous monitoring to ensure participant safety throughout the study.

### 2.6 Data management

#### 2.6.1 Completion and management of CRF

Researchers must accurately and thoroughly collect original data and use it to complete the CRF. To ensure strict adherence to the research protocol, the principal investigator will designate a clinical monitor to conduct regular supervisory visits to the research sites. Both paper and electronic research data must be stored for a minimum of five years, and biological specimens will be disposed of ethically after monitoring, in accordance with participants’ rights and interests.

#### 2.6.2 Data entry and modification

This study will collect participants’ electronic medical record information through an Electronic Data Capture (EDC) system. To ensure the accuracy of the data, data entry will be performed independently by two designated data entry clerks from each research unit, who will also proofread the data. For any queries in the Case Report Form, the data manager will fill out a Data Query Form (DRO) and send an inquiry to the researchers. The data manager will then modify the data based on the researchers’ responses.

#### 2.6.3 Data locking and review

Upon completion of data entry, the principal investigator and statistical analysts will lock the data while remaining blinded, after which no further modifications will be allowed. A line-by-line verification will then be conducted to review the entries made by both data entry personnel. A random sample of CRFs will be selected and cross-checked against the data in the EDC system to ensure consistency with the original source data.

### 2.7 Statistical analysis

Statistical analyses were performed using SAS version 9.3 (SAS Institute Inc., USA) and included baseline characteristics, efficacy, and safety outcomes. A p-value of < 0.05 was considered statistically significant. The efficacy analysis will follow the intention-to-treat (ITT) principle. The Full Analysis Set (FAS) includes all randomized patients, excluding those who severely violated the inclusion criteria or did not receive any assigned treatment. Baseline characteristics will be analyzed within this set to assess comparability. The Per-Protocol Set (PPS) includes patients who completed the planned treatment regimen, whereas the Safety Set (SS) consists of patients who received at least one dose of the study treatment and underwent safety evaluation. Efficacy analysis will be primarily applied to the FSA and PPS. Safety evaluations will be performed using the SS.

For primary and secondary outcomes, continuous data will be analyzed using the t-test or Mann-Whitney U test; categorical data will be assessed using the Chi-squared test or Fisher’s exact test; and ordinal data will be evaluated using the Mann-Whitney U test. To account for potential center effects in this multicenter trial, analyses will be stratified by study site. Efficacy will be evaluated using the Cochran-Mantel-Haenszel (CMH) test to adjust for inter-center variability.

If baseline characteristics between groups are imbalanced, confounding factors that are difficult to control or unmeasured may be included as covariates in an analysis of covariance (ANCOVA) or logistic regression model. Between-group differences in efficacy will be assessed using least-squares means (LSMEANs) and their 95% confidence intervals to minimize the impact of confounding factors.

### 2.8 Quality control

Researchers must strictly follow the study protocol and comply with standard operating procedures. All clinical observations and findings must be verified to ensure the effective implementation of quality control and quality assurance systems throughout the study. Quality control measures must be implemented at every stage of data processing to ensure the reliability and accuracy of the data.

Prior to study initiation, all participating researchers must undergo comprehensive training, including guidance on relevant data, operational procedures, and assigned responsibilities. Given that the study involves assessments using scales related to anxiety and depression, all researchers must be trained by a psychiatrist, and qualified professionals in psychological assessment must be included in the research team. This ensures consistency in clinical data collection and guarantees that all data are recorded objectively, accurately, and truthfully in the medical records and CRFs. Throughout the study, monitors from the lead research institution will conduct regular audits of source clinical data at study sites to ensure strict adherence to the study protocol. If a subject’s treatment regimen is modified during the study, investigators must record the details and reasons for the change and continue follow-up as planned. During data analysis, the principal investigator and statistical experts will allocate each subject to the appropriate analysis set.

Researchers should foster participant confidence in the treatment and maintain effective communication to ensure a clear understanding of the study requirements, thereby improving adherence. Participant adherence will be monitored using the medication count method. Before each medication dispensing, remaining study drugs and empty packaging will be collected. Adherence will be calculated as follows: [(prescribed amount - unused amount)/prescribed amount] × 100%. Good adherence: 80%-120%, Poor adherence: <80%.

Measures taken to reduce bias: Researchers must strictly adhere to the inclusion and exclusion criteria specified in the protocol, enroll participants consecutively, and maintain follow-up until the end of the study to minimize selection bias. Researchers should remind participants to take medication as prescribed, assess outcomes objectively, and strictly comply with laboratory procedures for sample testing to reduce information bias. Potential confounding factors should be addressed using stratified and multivariate analyses to reduce confounding bias.

## 3 Discussion

Depression may have a causal influence on the biological processes and behaviors associated with ACS, sharing several common pathological mechanisms. A bidirectional Mendelian randomization study has demonstrated a robust causal relationship between depression and an increased risk of coronary heart disease (21). Potential comorbid mechanisms include excessive immune-inflammatory response, activation of the HPA axis, endothelial dysfunction, and platelet hyperactivity (22). Depression activates the HPA axis, leading to hypercortisolemia, which is closely associated with the pathogenesis of cardiovascular diseases (23, 24). Individuals with depression often exhibit elevated levels of inflammatory markers including IL-1β, IL-6, TNF-α, and CRP, which are directly associated with acute cardiovascular events (24). Depression may also lead to immune-mediated pancreatic β-cell dysfunction, lifestyle alterations, and sustained activation of the HPA axis, resulting in visceral fat accumulation and central obesity. These factors promote insulin resistance and diabetes, both of which are significant risk factors for cardiovascular disease (25, 26). Depression has been associated with increased platelet thrombin reactivity, elevated platelet factor expression, reduced endothelial nitric oxide synthase activity, decreased serotonin transporter binding, and lower platelet serotonin levels. Enhanced platelet reactivity is believed to contribute to increased susceptibility to atherosclerotic thrombosis in individuals with depression (27). Among individuals with comorbid depression after ACS, secondary prevention strategies that prioritize mental health should be implemented. SSRIs, a class of antidepressants, are associated with numerous adverse effects. Additionally, certain antidepressants may lead to dependence and addiction, potentially resulting in poor adherence to treatment (18). Given the limited clinical treatment options, identifying safe and effective interventions for depression following ACS remains a critical challenge.

SGJY has been approved and widely prescribed in China for the treatment of depression since 2008 (20). A substantial body of clinical evidence (28–30) indicates that SGJY is more effective than conventional antidepressants and is associated with fewer adverse effects. Furthermore, preliminary studies suggest that SGJY may contribute to the prevention and treatment of coronary heart disease and exert cardioprotective effects: Extracts of *Hypericum perforatum* can reduce low-density lipoprotein cholesterol (LDL-C), alleviate oxidative stress and lipid peroxidation induced by hyperlipidemia, and are used in the prevention and treatment of atherosclerotic cardiovascular diseases. Hypericin (31), a major active constituent of *Hypericum perforatum*, has been shown to upregulate autophagy after myocardial infarction, thereby inhibiting the NLRP3 inflammasome pathway, alleviating myocardial hypertrophy, and reducing fibrinogen deposition, ultimately exerting cardioprotective effects. *Acanthopanax senticosus*, a constituent of SGJY, is widely used in the treatment of cardiovascular diseases. It protects the cardiovascular system by preserving ischemic myocardial cells, reducing reperfusion injury, inhibiting ventricular remodeling, and improving microcirculation (32). Extract of *Acanthopanax senticosus* also modulates the gut microbiota and lipid metabolism via regulation of the NF-κB signaling pathway, thereby contributing to anti-atherosclerotic effects (33). Epimedium E, another key bioactive compound in *Acanthopanax senticosus*, exhibits antioxidant, anti-inflammatory, and immunomodulatory activities. It inhibits inflammation and pyroptosis through the NLRP3/caspase-1 signaling pathway, thereby mitigating myocardial injury (34). Based on the above findings, we hypothesize that SGJY may exert beneficial therapeutic benefits in patients with depressive state after ACS. However, high-quality clinical evidence supporting this hypothesis remains limited. Therefore, we designed this trial to evaluate the clinical efficacy and underlying mechanisms of SGJY in the treatment of depressive state after ACS.

This study aims to assess the efficacy of SGJY in treating depressive state after ACS by examining series of clinical scales, inflammatory cytokines, and related proteins (IL-1β, IL-6, GDF15, and sCD40L). In addition, a 36-week follow-up will be conducted after SGJY treatment to record the incidence of MACCE and evaluate long-term prognosis. Clinical studies have shown that improvements in cardiac and pulmonary function reduce ischemic burden in patients with coronary artery disease (35), and that exercise capacity is a strong predictor of adverse cardiovascular events (36). Therefore, CPET will be included in the trial to assess patients’ cardiopulmonary endurance. Additionally, the study will measure serum levels of HPA axis-related hormones (CRF, ACTH, and cortisol) and BDNF. Upon activation of the HPA axis, the paraventricular nucleus of the hypothalamus synthesizes and releases more CRF. The CRF is transported to the anterior pituitary, where it stimulates the secretion of ACTH into the systemic circulation. ACTH acts on the zona fasciculata of the adrenal cortex promoting the synthesis and release of cortisol. HPA axis dysfunction and subsequent elevation of cortisol levels contribute to the progression of depression and an increased risk of adverse cardiovascular events (22). BDNF is a biological marker associated with depression and CHD. Reduced BDNF level is associated with an increased risk of depression in patients with ACS. Moreover, Meta-analyses have demonstrated an increase in BDNF after antidepressant treatment (37). Metabolomic analyses will be conducted to investigate the potential mechanisms through which SGJY exerts its antidepressant effects in patients with depression after ACS. GDF15 is a stress-responsive inflammatory cytokine primarily secreted by activated macrophages. Elevated GDF15 levels are closely associated with cardiovascular disease severity, increased risk of adverse cardiovascular events, and the presence of depressive symptoms (38–41). sCD40L serves as a diagnostic biomarker for atherosclerosis, a condition strongly associated with cardiovascular mortality and non-fatal myocardial infarction (42, 43). Serum sCD40L levels are also significantly elevated in individuals with depression, further increasing their risk of cardiovascular disease (44, 45).Therefore, GDF15 and sCD40L may serve as surrogate endpoints for predicting adverse cardiovascular events and may also reflect the severity of depressive symptoms.

This study has several limitations. First, the treatment period is limited to 12 weeks, with a follow-up visit scheduled 36 weeks after the end of treatment. This relatively short follow-up duration may restrict the ability to fully capture the occurrence of MACCE. This limitation elevates the risk of false-negative conclusions and consequently impairs the accuracy of evaluations regarding whether patients with ACS actually benefit from antidepressant treatment. However, previous studies have shown that biomarkers such as GDF15 and sCD40L are reliable surrogate indicators of long-term prognosis. Consequently, this trial may offer valuable insights into the potential role of SGJY in improving the prognosis of ACS. Second, CPET will be conducted by different operators across the five centers—a factor that introduces the risk of measurement variability and potential bias. Differences in test procedures, equipment calibration, or data interpretation among operators may compromise the accuracy of results and subsequent assessments of the intervention’s efficacy. To mitigate this risk, we implemented a stringent set of SOP for all CPET assessments. Furthermore, all operators and assessors received centralized, uniform training on operational and analytical protocols before the trial commenced. Third, the use of clinician-rated scales to evaluate treatment efficacy may lead to subjective bias. To ensure the reliability of the evaluation results, all researchers will undergo standardized training provided by psychiatrist before the study get started. In addition, objective biomarkers (including inflammatory cytokines, relevant proteins, HPA axis-related hormones, and BDNF) will be incorporated to support efficacy evaluation. Fourth, The use of Consensus criteria in this study may introduce over-diagnosis bias by potentially including patients who meet diagnostic criteria for Major Depressive Disorder (F32.0/F32.1) or Organic Depressive Disorder (F06.32). To address this limitation, we will perform stratified analyses by diagnostic subgroup to assess result reliability.

## 4 Conclusion

This multicenter, randomized, double-blind, placebo-controlled clinical trial is designed to evaluate the efficacy of SGJY in treating depressive state after ACS, thereby providing a foundation for high-quality, evidence-based assessments of SGJY. The long-term follow-up component, which monitors MACCE, aims to provide evidence supporting the potential role of SGJY in improving long-term outcomes in patients with depressive state after ACS.
